# Epidemiological and spatiotemporal analysis of severe fever with thrombocytopenia syndrome in Eastern China, 2011–2021

**DOI:** 10.1186/s12889-023-15379-3

**Published:** 2023-03-16

**Authors:** Shuyi Liang, Zhifeng Li, Nan Zhang, Xiaochen Wang, Yuanfang Qin, Wei Xie, Changjun Bao, Jianli Hu

**Affiliations:** 1 Acute Infectious disease control and prevention institute, Jiangsu Provincial center for disease control and prevention, Nanjing, China; 2 Institute of Food Safety and Assessment, Jiangsu Provincial center for disease control and prevention, Nanjing, China

**Keywords:** Severe fever with thrombocytopenia syndrome (SFTS), Epidemiology characteristic, Space-time scanning, Clustering

## Abstract

**Background:**

Severe fever with thrombocytopenia syndrome (SFTS) is an emerging infectious disease, which is caused by severe fever with thrombocytopenia syndrome virus (SFTSV) with high fatality. Recently, the incidence of SFTS increased obviously in Jiangsu Province. However, the systematic and complete analysis of spatiotemporal patterns and clusters coupled with epidemiological characteristics of SFTS have not been reported so far.

**Methods:**

Data on SFTS cases were collected during 2011–2021. The changing epidemiological characteristics of SFTS were analyzed by adopting descriptive statistical methods. GeoDa 1.18 was applied for spatial autocorrelation analysis, and SaTScan 10.0 was used to identify spatio-temporal clustering of cases. The results were visualized in ArcMap.

**Results:**

The annual incidence of SFTS increased in Jiangsu Province from 2011 to 2021. Most cases (72.4%) occurred during May and August with the obvious peak months. Elderly farmers accounted for most cases, among which both males and females were susceptible. The spatial autocorrelation and spatio-temporal clustering analysis indicated that the distribution of SFTS was not random but clustered in space and time. The most likely cluster was observed in the western region of Jiangsu Province and covered one county (Xuyi county) (Relative risk = 8.18, Log likelihood ratio = 122.645, *P* < 0.001) located in southwestern Jiangsu Province from January 1, 2017 to December 31, 2021. The Secondary cluster also covered one county (Lishui county) (Relative risk = 7.70, Log likelihood ratio = 94.938, *P* < 0.001) from January 1, 2017 to December 31, 2021.

**Conclusions:**

The annual number of SFTS cases showed an increasing tendency in Jiangsu Province from 2011 to 2021. Our study elucidated regions with SFTS clusters by means of ArcGIS in combination with spatial analysis. The results demonstrated solid evidences for the orientation of limited sanitary resources, surveillance in high-risk regions and early warning of epidemic seasons in future prevention and control of SFTS in Jiangsu Province.

## Background

Severe fever with thrombocytopenia syndrome (SFTS) is an emerging infectious disease caused by the SFTS virus (SFTSV). It was firstly discovered in China in 2009 [1]. The vast majority of SFTS was indentified in Shandong, Hubei, ,Henan Anhui, Liaoning, Zhejiang and Jiangsu Provinces. Of note, over the last decade and more in China, the number of SFTS cases not only continued to increase but also had an expansion of geographical distribution. In recent years, more than 15 provinces in China have reported SFTS [2]. However, SFTS cases were also indentified in other countries, such as Japan, South Korea and Vietnam [3–7]. SFTSV infection had also been first reported in Myanmar, Taiwan, Thailand and Pakistan in 2020 and 2022 [8–11].

The main clinical manifestation of SFTS included high fever, leukopenia and thrombocytopenia, and multiple organ [12], with a mortality rate of more than 10% [13, 14]. SFTSV was mainly transmitted through tick bites. Besides, SFTSV could also spread from person to person via direct contact with blood [15]. What is more, the persons infected with SFTSV by direct contact with the dogs and cats had been discovered, which become a public health concern [16, 17]. Due to the life-threatening threat to public health, SFTS was chosen as one of the 9 emerging diseases given a priority for research and development by the World Health Organization in 2017 [18].

The predominant vector of SFTS is *Haemaphysalis longicornis* (*H. longicornis*) whose distribution is closely associated with the meteorological factors accompanied by remarkable seasonal features. Along with the variation of these factors, the occurrence and spread of SFTS vary over space and time. Therefore, the widespread adoption of geographic information systems and spatial statistics in describing the distribution characteristics and transmission patterns of diseases might not only be conducive to timely surveillance, but also to effective intervention against infectious diseases.

However, so far, there are few studies on spatial epidemiological characteristics at the county level in Jiangsu Province. Tick-borne diseases have different spatial and temporal distribution characteristics in different regions, which is affected by factors such as meteorologic factors, social and economic condition and geographical location [19, 20]. Previous studies have shown that the spatial distribution of tick-borne diseases within a country or even on a sub-national scale has considerable [21]. As the detection technology, increasing awareness, and the change of environmental factors develop, an increasing number of SFTS cases were reported in Jiangsu Province. Hence, the objective of the study was to summarize the changing epidemiological characteristics of SFTS in Jiangsu Province and observe whether some characteristics have changed. Furthermore, the heterogeneity of spatial-temporal of SFTS was analyzed at the county level, which would provide a theoretical knowledge basis for its accurate and scientific control and prevention.

## Methods

### Study area

Jiangsu is a coastal province located in the middle of east coast of China (30°45’- 35°08’ N, 116°21’ − 121°56’ E), featuring a monsoon climate transitioning from a subtropical to a temperate one (Fig. 4). The landscape of Jiangsu Province includes plains, mountains and hills. County or district is taken the study unit and Jiangsu Province is administratively divided into 95 counties or districts.

### Case definition

One patient who had a fever (≥ 38.0℃) and presented other symptoms (e.g. digestive symptoms, bleeding) also exhibiting risk factors related to epidemiology (ticks exposure two weeks before illness onset or working as a farmer) and clinical testing data included thrombocytopenia and leukocytopenia, was considered a suspected case. The suspected patient meeting one or more of the following conditions: [1] SFTSV RNA identification; [2] seroconversion or 4-fold increase in antibody titers between paired serum samples collected at two-week or more intervals; and [3] the SFTSV isolated from cell culture considered as a confirmed case [22].

### Data sources

The data of SFTS cases between January 2010 and December 2021 in Jiangsu Province were obtained from the Nationwide Notifiable Infectious Diseases Reporting Information System (NIDRIS). Every case was identified by laboratory or clinical diagnosis. Information including the residential address, age, gender, occupation and date of illness onset of the SFTS cases was contained. In addition, the demographic information was obtained from Jiangsu statistical annual report (tj.jiangsu.gov.cn/col/col87172/index.html).

### Statistical analysis

IBM SPSS Statistics for Windows, version 25.0 (IBMCorp., Armonk, N.Y., USA) was used for analyzing and processing basic data, while *ArcGis* 10.7 (ESRI, Redlands, CA, USA) was applied for the visualization of geographical distribution.

### Spatial autocorrelation analysis

Besides, global spatial autocorrelation analysis and local autocorrelation analysis were conducted using GeoDa1.18 software, and Global Moran’s I was used to explore the spatial pattern of the whole study area, the [− 1,1] range of Global Moran’s *I*, and how closer its value is to 1. The closer the relationship between the spatial units is, the more similar their properties become, and conversely, the closer it is to − 1, the greater the difference between the spatial units [23] will be. Z statistics and values were consistent with the results of Monte Carlo tests (999 times), and the main spatial weight matrix was set by the Queen method.

When spatial heterogeneity is defined as the presence of significant variation in spatial autocorrelation within the study area, it is necessary to describe the variation of spatial autocorrelation in the study area. Herein, local indicators of spatial association were depicted by local indicators of spatial autocorrelation (LISA) [24], and were classified into five categories on the LISA Map, i.e., High-High; Low-Low; None (spatial outliers not significant); High-Low; and Low-High.

### Space-time cluster analysis

SaTScan10.0 software and *ArcGIS10.7* were used for spatio-temporal scanning analysis and spatio-temporal clustering visualization, respectively, while the clusters of temporal aggregation was monitored using spatial scan statistics. Additionally, the analysis was extended to three-dimensional space-time cluster by Kulldorff, which had been widely used in epidemiology and other fields [25]. The statistical principle of spatio-temporal scanning was to create a cylindrical scanning window with the study area as the base, the time as the height, and the population at risk as the radius. The risk in each scanning window was examined, and the likelihood ratio (LLR) was also calculated. The value of LLR in different windows was calculated and could be considered as statistics to be compared and tested by Monte-Carlo sampling. The *P*-value was obtained by Monte Carlo randomization, and *P* < 0.05 was considered as spatio-temporal aggregation.

Herein, the maximum spatial scanning radius was set as 50% of the risk population, and the scanning time ranged from January 1, 2011 to December 31, 2021. The time interval was set to ‘Year’ and the region was also set to ‘None’.

### Ethics statement

This present research reported here has been approved by the Ethics Committee of Jiangsu Provincial CDC.

## Results

### Epidemiological characteristics

**Fig. 1:**
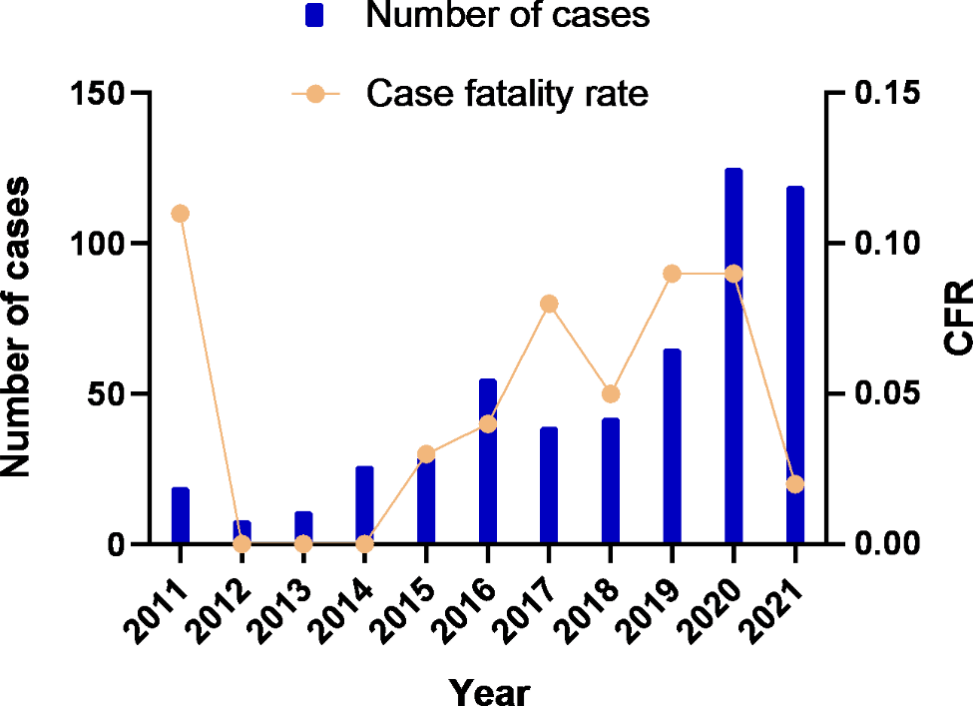
The number of severe fever with thrombocytopenia syndrome (SFTS) cases and case fatality rate in Jiangsu Province from 2011 to 2021

A total of 538 confirmed SFTS cases were reported from 2011 to 2021 in Jiangsu Province. The incidence interval ranged from 0.020/100,000 (2011) to 0.140/100,000 (2021), with an annual average incidence of 0.059/100,000. The annual case was 19, 8, 11, 26, 29, 55, 39, 42, 65, 125 and 119, presenting an increasing tendency from 2011 to 2021 (Fig. 1). The difference of annual case in different years was significant (χ^2^ = 31.02, *P* < 0.05) and the average case fatality rate (CFR) was 5.39% (29/538).

**Fig. 2:**
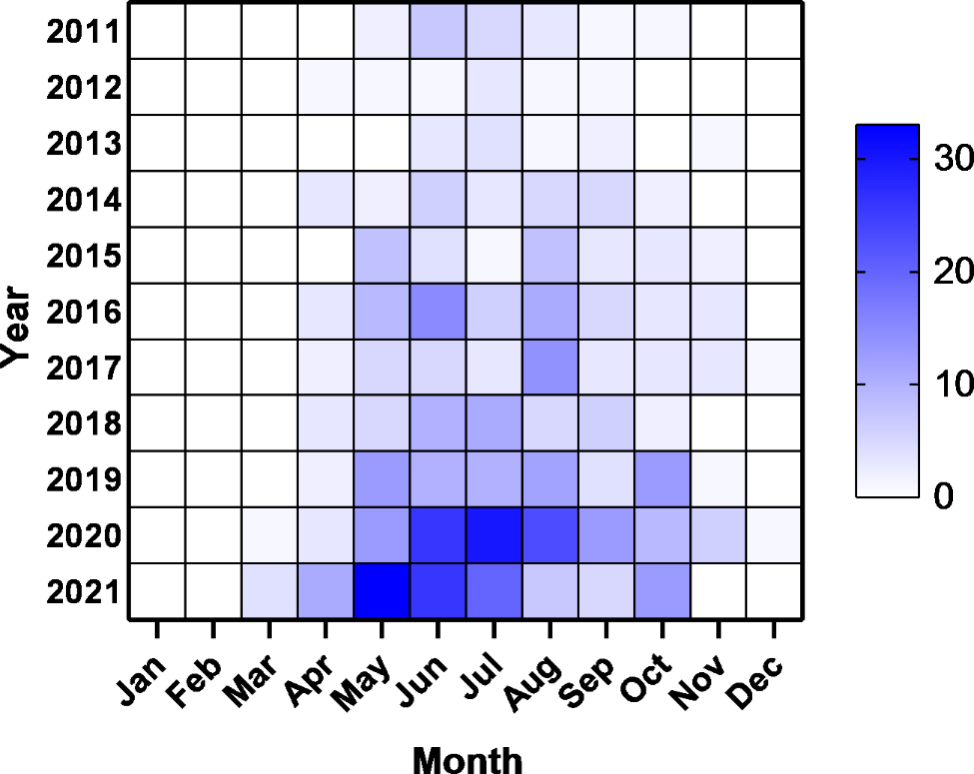
Monthly distribution of SFTS cases in Jiangsu Province from 2011 to 2021

The time distribution of the reported cases indicated obvious seasonal characteristics. The heat map depicted the monthly distribution of SFTS cases in Jiangsu Province from 2011 to 2021. A total of 390 cases (72.49%) occurred during May and August. Additionally, the peak of incidence in 2021 was earlier than that in previous years (Fig. 2).

**Fig. 3:**
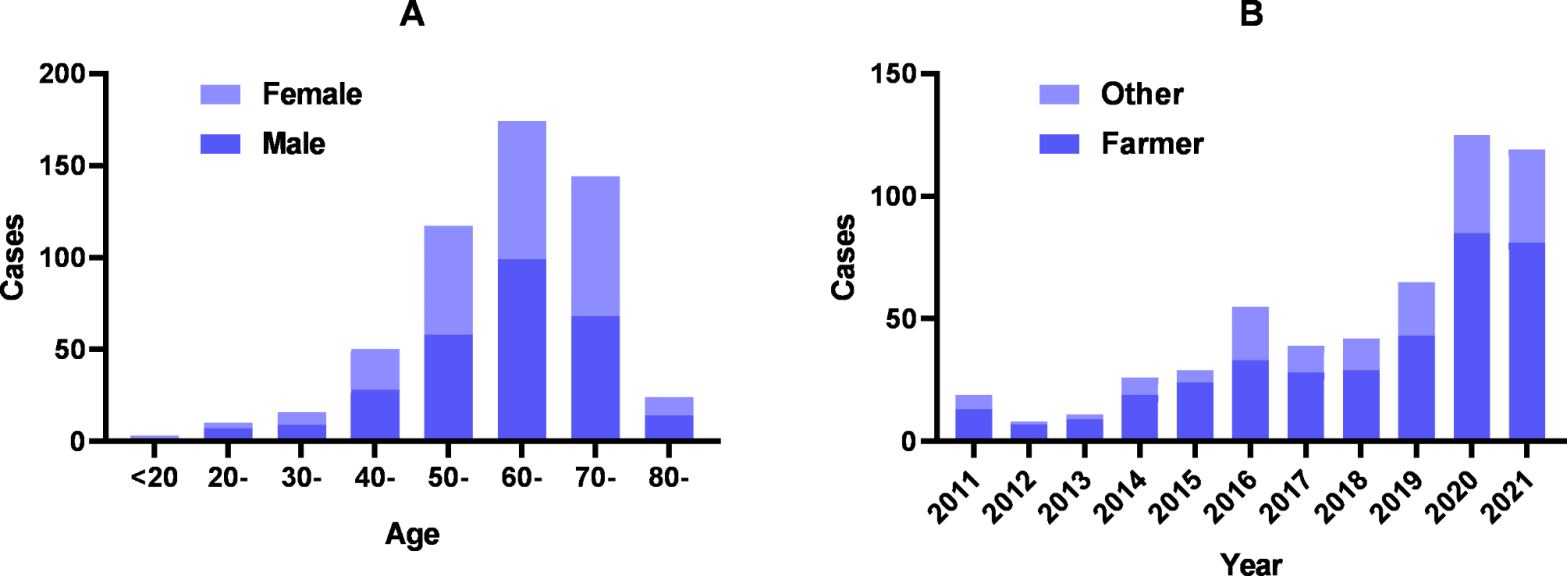
Gender-age and occupation distribution of SFTS cases in Jiangsu Province from 2011 to 2021

Of the 538 cases, 284 were males and 254 were females (the male-to-female ratio was about 1.118). The age distribution was mainly between 50 and 70 years old, accounting for 80.85% of all cases. However, there was no significant difference (χ^2^ = 5.6, ,*P >* 0.05) between males and females in all age groups(Fig. 3A). Of the 538 SFTS cases, 371 cases (68.96%) were farmers, accounting for the largest proportion **(**Fig. 3B**)**.

**Fig. 4:**
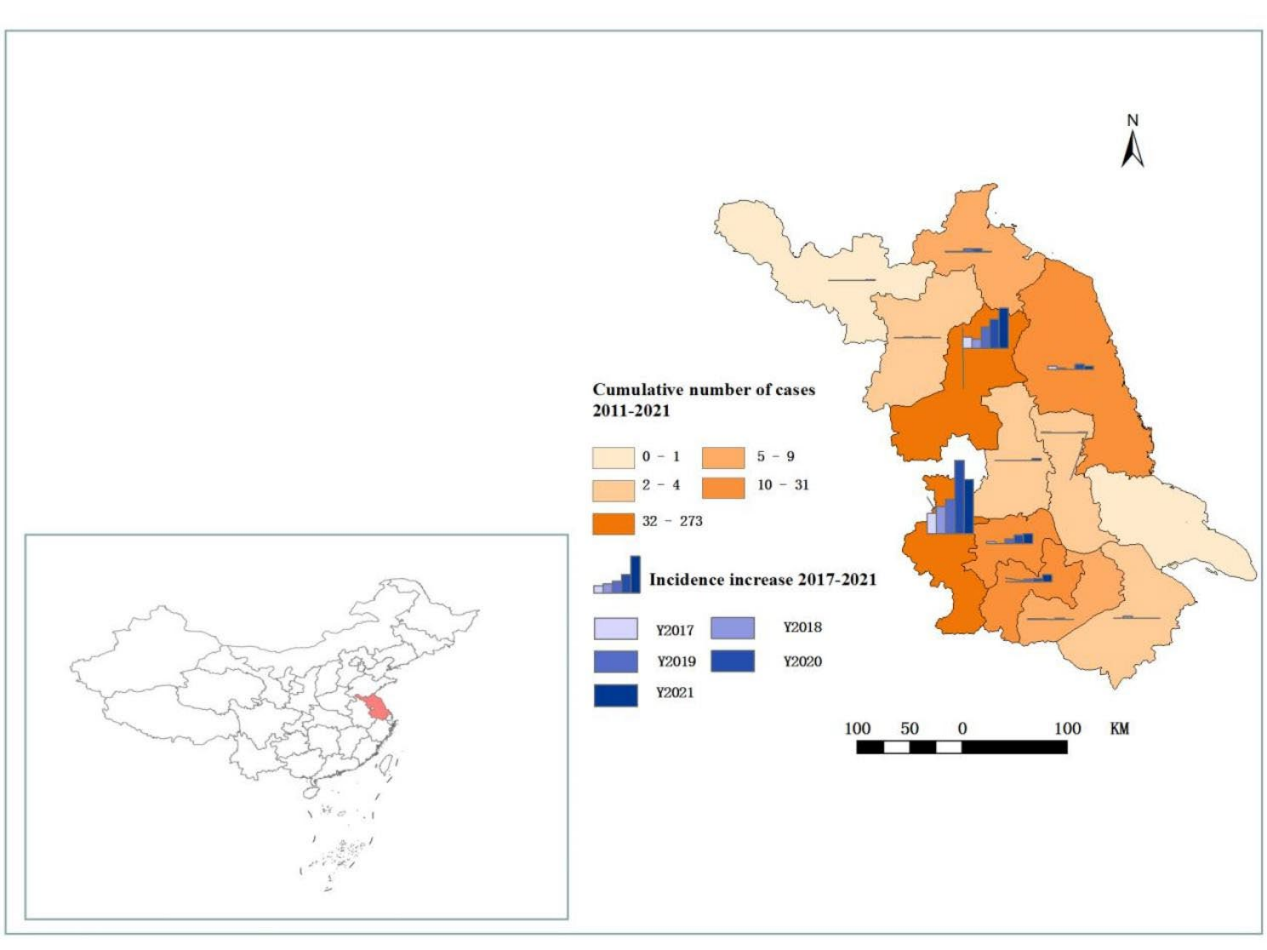
The location of Jiangsu Province and the region distribution of SFTS cases in Jiangsu Province from 2011 to 2021

The SFTS cases in Jiangsu Province were distributed in 12 districted cities from 2011 to 2021, mainly in Nanjing City, Huai ‘an City and Zhenjiang City. In the recent 5 years, the number of SFTS cases in each districted city showed an increasing trend year by year (Fig. 4). The numbers of SFTS affected counties from 2011 to 2021 were 6, 5, 6, 11, 8, 17, 13, 14, 14, 19, 18, respectively. A total of 38 counties had been affected by SFTS. Notably, the epidemic focus was getting increasingly larger. The three most seriously affected areas were Xuyi County of Huai’an City, Lishui County of Nanjing City and Jiangning County of Nanjing City, where the numbers of identified cases were 146, 106 and 51, respectively from 2011 to 2021.

### Spatial autocorrelation analysis

**Fig. 5:**
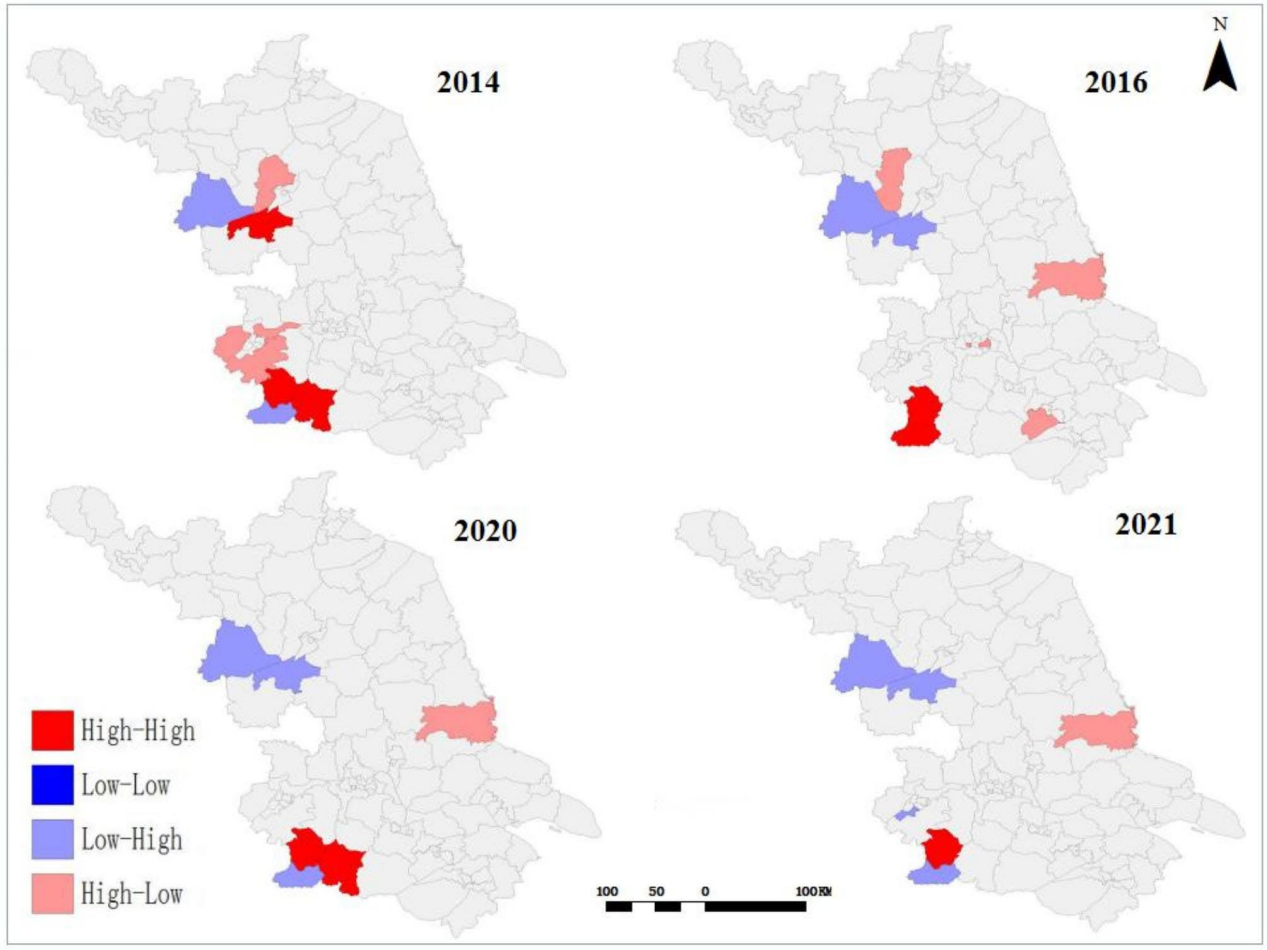
LISA Cluster Map in Jiangsu Province during 2011 to 2021

The Moran’s *I* by global spatial autocorrelation analysis showed positive global spatial autocorrelations (Moran’s *I* = 0.106 in 2014, 0.133 in 2016, 0.097 in 2020, and 0.085 in 2021), indicating the positive correlation property of the regional distribution of SFTS cases (Table 1). The results of local spatial autocorrelation showed that the High-High clusters were mainly located in adjacent areas including Lishui County, Gaochun County and Liyang County (Fig. 5).

**Table 1:** The Moran’s *I* of global spatial autocorrelation analysis during 2011 to 2021

Year	Moran’s *I*	*Z* score	*P-*value
2011	−0.00	0.25	0.24
2012	0.061	2.02	0.08
2013	0.001	0.33	0.24
2014	0.106	2.77	0.02
2015	0.022	0.97	0.10
2016	0.133	3.13	0.02
2017	0.080	1.93	0.05
2018	0.069	1.57	0.07
2019	0.058	1.53	0.07
2020	0.097	2.39	0.03
2021	0.085	2.27	0.04

### Space-time cluster analysis

**Fig. 6:**
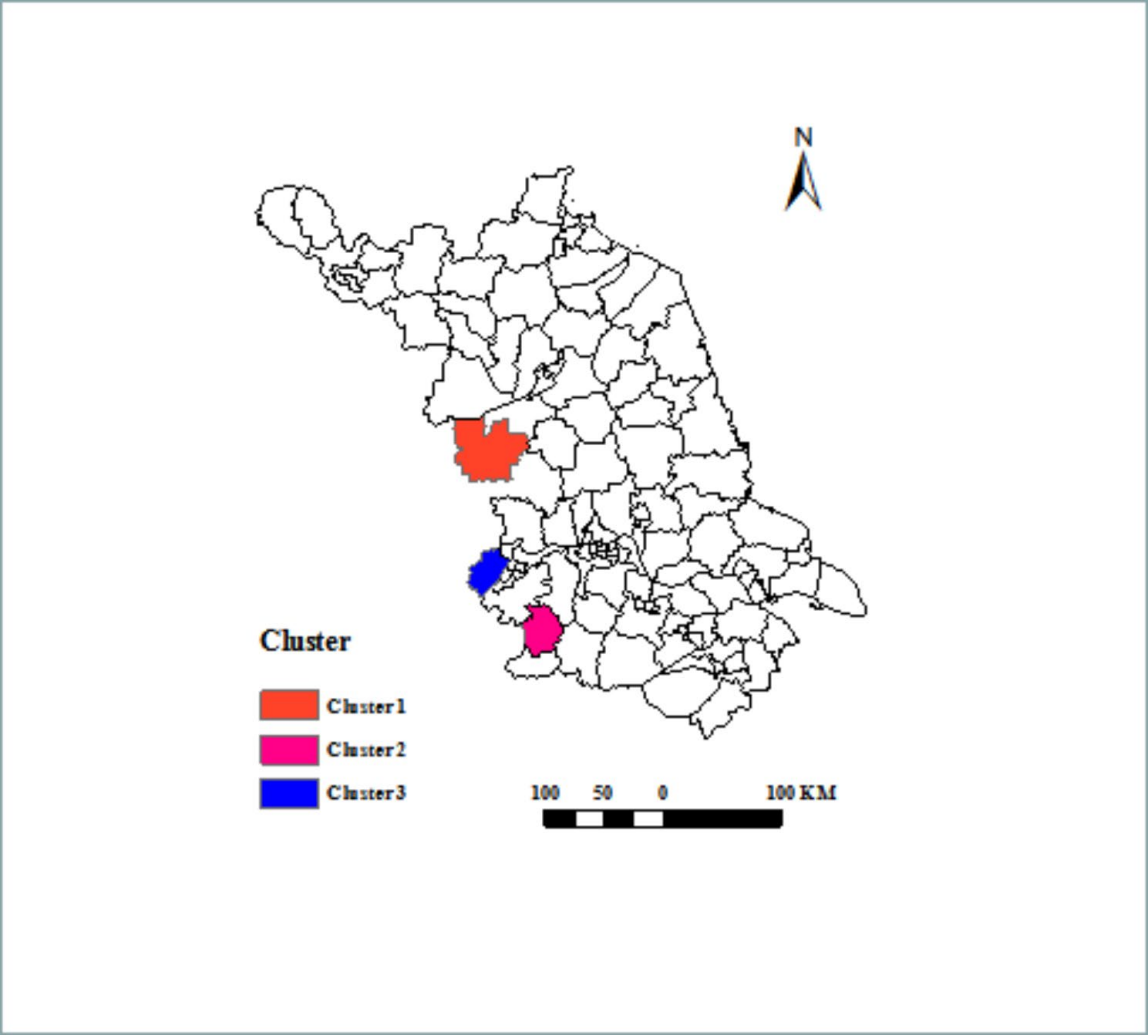
Time-space clusters of SFTS cases at county level in Jiangsu Province from 2011 to 2021

As shown in Table 2 Fig. 6, the most likely cluster was observed in the western region of Jiangsu Province, covering one county (Xuyi County) (Relative risk = 8.18, Log likelihood ratio = 122.645, P < 0.001) located in southwestern Jiangsu Province from January 1, 2017 to December 31, 2021. The secondary cluster also covered one county (Lishui County) (Relative risk = 7.70, Log likelihood ratio = 94.938, P < 0.001) from January 1, 2017 to December 31, 2021. Additionally, the tertiary cluster was Pukou County (Relative risk = 4.74, Log likelihood ratio = 28.292, P < 0.001) from January 1, 2018 to December 31, 2021.

**Table 2:** The results of spatial-temporal scanning

Cluster	Primary Cluster 1	Secondary Cluster 2	Tertiary Cluster 3
Location	Xuyi	Lishui	Pukou
Coordinates / radius	(32.969318 N, 118.526272 E) / 0 km	(31.596370 N, 119.028989 E) / 0 km	(32.039690 N, 118.537480 E) / 0 km
Time frame	2017/1/1 to 2021/12/31	2017/1/1 to 2021/12/31	2018/1/1 to 2021/12/31
Population	607,215	491,334	364,826
Number of cases	108	86	38
Expected	16.05	12.99	8.49
Annual cases (1/100,000)	3.6	3.5	2.4
Relative risk	8.18	7.70	4.74
LLR	122.645	94.938	28.292
*P* value	< 0.001	< 0.001	< 0.001

## Discussion

Jiangsu Province is located in the core area of the beautiful and rich Yangtze River Delta of China, whose the terrain is mainly plains and hills. In this study, we analyzed the changing epidemiological characteristics of SFTS cases in Jiangsu Province between 2011 and 2021. We found an annual average incidence of 0.059/100,000. The average incidence of SFTS in Jiangsu Province was lower than that in Shandong province in China [26]. We also elucidated that the number of annual SFTS cases and affected counties increased year by year. Some factors may be associated with the results. The reason for the spatial expansion of the disease might be attributed to the border-crossing transportation of domestic animals that carried ticks and led to the infection. In recent years, the ecological environment in Jiangsu Province has changed, and become more suitable for the survival of vector ticks of SFTSV in nature. In addition, not only the improvement of diagnosis capacity but also the enhanced detection techniques may contribute to the increasing number of reported SFTS cases.

The SFTS in Jiangsu Province were highly seasonal. The heat map of the monthly distribution indicated that the epidemic season spanned from May to August, which was similar to the results found in a previous study [24, 27–30]. The epidemic season appeared to be slightly later at a higher latitude [21]. The seasonal distribution feature might be associated to tick dynamics. *Haemaphysalis longicornis*, as the predominant population carrying SFTSV [31, 32], the ecological characteristic was also in agreement with the corresponding results. The peak months in different years were [12]. Of interest, the SFTS cases reached a peak in May in 2021, which was earlier than that in any previous year. The reason for the May 2021 peak might relate to people’s activities in that year due to the impact of the COVID-19 pandemic. People prefered to go camping outside since spring and therefore had more opportunities to expose themselves to SFTSV compared to previous years. Thus, more attentions needed to be paid in the following years.

We also found that the majority of the cases were farmers. The results were in accordance with the previous studies [33]. What is more, most cases were in the elderly. Due to the decrease in immunity and underlying medical conditions, the elderly might be more likely infected by SFTSV. Another factor may also contribute to the result. In these affected counties, the majority of cases were elderly farmers living in hilly areas as young people went to cities to earn more money. Therefore, the elderly farmers had more chance of exposing themselves to ticks. These results may be helpful for authorities to better preventive strategies and improve interventions against SFTS.

By using the global Moran’s *I* index to detect whether the positive spatial autocorrelation existed and using LISA to explore the high-risk hotpot, our observations shed light on gathering centers, which were mainly concentrated in Lishui County and its adjacent areas including Gaochun County and Liyang County. Of note, the high-risk areas were relatively fixed without expanding tendency for now. Meanwhile, the results of the spatiotemporal clusters analysis indicated that the gathering areas were concentrated in Xuyi County, Lishui County and Pukou County. Overall, the concentrated areas of SFTS in Jiangsu Province were mainly in Xuyi County and Lishui County by combining two methods of analysis. In these gathering areas, there were a lot of mountains and hills with a high density of ticks. Therefore, local people or people travelling to these areas could get more chances of SFTSV infection. What’s more, several other factors such as meteorological factors, birds migration and SFTSV-carrying rate of ticks might be also associated with SFTS occurrence in these gathering areas [19, 27, 34–37]. Hence, an additional study is warranted to elucidate the influence of these factors on SFTS incidence in these natural epidemic foci.

However, this study had a few limitations. Firstly, the datas obtained from NIDRIS might be underestimated because of underreporting. For example, those who did not go to the hospital for treatment, or those who had died before being sent to the medical facility, were not included. Second, due to the time interval set “year”, the spatiotemporal scanning analysis might hide the monthly local SFTS clusters.

## Conclusion

This present study demonstrated epidemiological characteristics and spatiotemporal patterns of SFTS in Jiangsu Province from 2011 to 2021. Our results revealed that the annual number of SFTS cases and the affected counties had an increasing trend in Jiangsu Province. SFTS cases concentrated from May to August. Moreover, our study clearly elucidated that the distribution of SFTS was not random but clustered in space and time, with high-high clusters located in the western and southwestern areas of Jiangsu Province, China. Overall, our study threw light on epidemiological and spatial clues for finding out the factors of influence related to the incidence of SFTS. Meanwhile, the results provided solid evidence for the orientation of limited sanitary resources, surveillance in high-risk regions and early warning of epidemic seasons in future prevention and control of SFTS in Jiangsu Province.

## Data Availability

Data used in this present study are presented within the article.
